# Novel and Effective Blood‐Based miRNA Diagnostic Panel for Gastric Cancer: A Pilot Study in a Japanese Population

**DOI:** 10.1002/cam4.70790

**Published:** 2025-04-18

**Authors:** Tatsuro Murano, Ka Yan Chung, Yew Chung Tang, Yuki Kano, Ken Takeuchi, Naoya Sakamoto, Takeshi Kuwata, Yuan Gao, Jit Kong Cheong, He Cheng, Lihan Zhou, Tomonori Yano

**Affiliations:** ^1^ Department of Gastroenterology and Endoscopy National Cancer Center Hospital East Chiba Japan; ^2^ MiRXES Lab Pte Ltd. Singapore; ^3^ Department of Gastroenterology Tsujinaka Hospital Kashiwanoha Chiba Japan; ^4^ Department of Pathology National Cancer Center Hospital East Chiba Japan; ^5^ Department of Genetic Medicine and Services National Cancer Center Hospital East Chiba Japan

**Keywords:** blood, early detection of cancer, gastric cancer, liquid biopsy, miRNA

## Abstract

**Background:**

Gastric cancer (GC) has a high prevalence in Asian countries, and there is an unmet need for non‐invasive and efficient GC screening methods. This study evaluated the diagnostic efficacy of GASTROClear, a panel of blood serum miRNAs for the detection of GC, in a Japanese population.

**Methods:**

We conducted a pilot cohort study, comprising 103 patients with GC and 122 healthy controls. Serum samples were prospectively collected from study participants at two Japanese hospitals using a predefined blood‐processing protocol. The diagnostic performance of GASTROClear was analyzed using a receiver operating characteristic curve and cutoff. By applying a logistic regression algorithm, we evaluated the diagnostic efficacy of novel combinations of GC diagnostic biomarker panels, consisting of GASTROClear and alternative serum markers (anti‐ *Helicobacter pylori* [Hp] antibody and pepsinogen).

**Results:**

Most patients had Stage I (58%) GC and were asymptomatic (59%). The area under the curve (AUC) value for the detection of GC using GASTROClear was 0.80, with 70.9% sensitivity and 75.2% specificity. GASTROClear performed equally well within the subgroups based on age, sex, symptoms, Hp status, and tumor characteristics. We improved the diagnostic performance of GASTROClear by combining it with an anti‐Hp antibody and pepsinogen. This yielded an AUC value of 0.88, with the highest specificity (86.9%) at a fixed sensitivity (70.9%).

**Conclusions:**

GASTROClear demonstrated competent diagnostic efficacy for GC in the detection of GC in our Japanese cohort, even in the early stages of cancer and asymptomatic cases. Its combination with existing serum markers may contribute to efficient risk stratification to detect GC.

## Graphical abstract

GASTROClear, a blood serum miRNA panel, shows promise for early gastric cancer detection in a Japanese population. Combined with other serum markers, it enhances diagnostic accuracy.

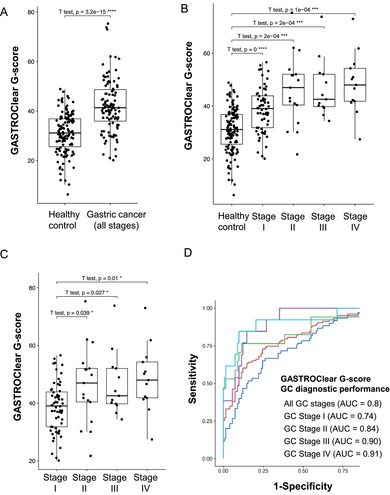

**Abbreviations**
AJCC: American Joint Committee on Cancer
AUC: area under the curve
CA: carbohydrate antigen
CEA: carcinoembryonic antigen
CI: confidence interval
GC: gastric cancer
HC: healthy controls
Hp:
*Helicobacter pylori*

IFU: instructions for use
PepI: pepsinogen I
PepII: pepsinogen II
qPCR: quantitative polymerase chain reaction
ROC: receiver operating characteristic
RT‐qPCR: reverse transcription quantitative polymerase chain reaction

## 1 Introduction

Gastric cancer (GC) is the fifth most commonly diagnosed cancer worldwide, and it is particularly prevalent in Asia [1]. The prognosis largely depends on the clinical stage of GC at the time of diagnosis. Cases that are detected early may be cured by endoscopic or surgical resection, whereas advanced cases with distant metastases often indicate a poor prognosis [2]. Since most patients with early‐stage cancer are asymptomatic, GC is often diagnosed at an advanced stage.

In Japan and South Korea, the implementation of nationwide mass screening using gastrointestinal endoscopy has been effective in reducing GC‐related mortality [3, 4]. The 5‐year survival rate of GC in countries with similar screening programs is extremely favorable when compared to countries without GC mass screening programs [1, 5]. Although gastrointestinal endoscopy is the standard of care for early‐stage GC detection, it may not be a cost‐effective mass‐screening tool in countries with low GC prevalence. Moreover, to achieve this reduction in mortality due to endoscopic screening, there must be sufficient endoscopy capacity coupled with a high uptake rate [6, 7]. Only a limited number of regions in Japan have sufficient endoscopic capacity [8]. Additionally, the anxiety, pain, and complications associated with endoscopy render compliance challenging, even in countries with a high prevalence of GC [9]. There is an unmet need to develop a minimally invasive and affordable diagnostic modality for the early detection and risk stratification of the population to identify individuals at high risk of GC.

Recently, GASTROClear, a molecular assay comprising a panel of serum miRNA biomarkers, was developed to enable minimally invasive diagnosis of GC [10]. The GASTROClear miRNA biomarker panel includes 12 miRNAs that have been implicated to be oncogenic or tumor suppressive and regulate various hallmarks of cancer, such as sustaining cell proliferation, evading tumor suppressors, activating invasion and metastasis, inducing angiogenesis, and resisting cell death (Table S1). This panel is based on a highly sensitive and reproducible reverse transcription quantitative polymerase chain reaction (RT‐qPCR) assay, where its diagnostic value for GC has been demonstrated in symptomatic patients from a large‐scale, prospective study conducted in Singapore [10]. Consequently, GASTROClear has been approved for clinical use in Singapore and Europe.

In this study, we conducted a pilot multicenter trial involving patients with GC and healthy participants in Japan to evaluate the diagnostic efficacy of GASTROClear. In particular, we aimed to assess the diagnostic performance of GASTROClear for early‐stage and asymptomatic GC, which could not be fully evaluated in the Singapore cohort and the reproducibility of its diagnostic capability across different Asian ethnicities.

## 2 Materials and Methods

### 2.1 Study Design and Population

The original GASTROClear assay was developed based on a three‐phase, multicenter study comprising 5248 subjects from Singapore and Korea [10]. To conduct a pilot study validating the clinical utility of the GASTROClear assay and exploring further optimization, we prospectively collected blood samples from 123 patients with GC and 132 healthy controls (HC) from two hospitals in Japan. Between April 2020 and January 2022, patients diagnosed with GC who were referred to the National Cancer Center Hospital East for treatment and HC who visited the Tsujinaka Hospital Kashiwanoha or National Cancer Center Hospital East for routine medical checkups were invited to participate in the study. Participants were assigned to the GC group if they were treatment‐naïve and pathologically diagnosed with adenocarcinoma by endoscopic biopsy. Participants were assigned to the HC group if they were confirmed to have no GC based on endoscopic and histological examinations and no abnormal findings from chest radiography, fecal occult blood tests, and abdominal ultrasound examinations. Based on the instructions for use (IFU) of GASTROClear, 20 GC and 10 HC samples were excluded from the analysis based on the following criteria: (1) under the age of 40 years, (2) samples with hemolysis, (3) samples with clotting time values outside the specified range, and (4) a history of cancer or concomitant cancer. The study protocol was approved by the respective institutional review boards of each hospital (2019‐161 at the National Cancer Center Hospital East and 2020‐5 at Tsujinaka Hospital Kashiwanoha). All the participants provided written informed consent.

### 2.2 Definition of Clinicopathological Characteristics During Enrollment

All patients with GC were classified according to the TNM staging system (UICC version 8) [11]. Histological diagnosis was performed by two independent pathologists in accordance with World Health Organization criteria [12]. Histological typing of GC was performed using specimens from endoscopic resection or surgery and biopsy specimens for cases treated with chemotherapy. When multiple histological types were mixed in a single pathological specimen, the predominant histological type within an area was used. Patients were considered asymptomatic for GC if GC was detected using gastric radiography or endoscopy screening during medical checkups or routine examinations. Patients were considered symptomatic for GC if GC was detected using endoscopy when investigating the cause of any symptoms or abnormal blood test results. The degree of atrophic mucosa was evaluated endoscopically and classified according to the Kimura–Takemoto classification [13]. An atrophic status rated as C‐2/3 or O‐1/2/3 was considered atrophic gastritis.

### 2.3 Sample Collection and Serum Processing

Whole‐blood samples were collected using the Venoject II vacuum serum collection tubes (VP‐AS109KM60; TERUMO, Tokyo, Japan). The tubes were gently inverted 10 times, followed by incubation in an upright position at room temperature for 30–90 min to allow the samples to clot. After clotting, the tubes were centrifuged for 15 min at 1500 RCF at room temperature. Subsequently, the supernatant was carefully aspirated from the surface of the liquid using a pipette and aliquoted into cryogenic vials. The vials were immediately stored at −80°C. Serum samples were prepared at the respective recruitment sites according to the same study protocol. All serum samples were shipped to the MiRXES Lab within 6 months after collection for GASTROClear analysis.

### 2.4 GASTROClear Profiling

Sample profiling using GASTROClear was performed in accordance with the IFU. Briefly, total RNA was extracted from 200 μL of serum from each participant and reverse transcribed to cDNA prior to its use in quantitative polymerase chain reaction (qPCR). qPCR was used to quantify the GASTROClear miRNAs in a single‐plex format. A numerical GC risk score (GASTROClear G‐score) for each sample was generated using GASTROSmart software (MiRXES, Singapore). The GASTROClear G‐score was calculated using a linear regression model of the measured expression levels of 12 miRNAs in the panel, based on the most optimal sensitivity and specificity combination. A score of 40 or more was defined as a positive test result [10]. GASTROClear profiling was performed without access to any clinical information.

### 2.5 Other Blood‐Based GC Marker Assays

Serum anti‐ *Helicobacter pylori* (Hp) IgG antibody (Hp‐Ab) levels were determined using ELISA according to the manufacturer's instructions (Denka Company Limited, Tokyo, Japan). Based on the manufacturer's criteria, an Hp‐Ab value of 10 or higher was considered positive. Carcinoembryonic antigen (CEA) and carbohydrate antigen (CA) 19‐9 were assessed by ELISA (SRL, Tokyo, Japan). Serum pepsinogen I (PepI) and II (PepII) levels were measured using human PepI and PepII ELISA kits (Sigma‐Aldrich, St. Louis, MO, USA). The PepI/PepII ratio was calculated.

### 2.6 Statistical Analysis

To compare the *G*‐scores between GC and HC groups, as well as among the different GC stages, we employed two‐sample *t*‐tests. The performance of the GASTROClear, Hp‐Ab, pepsinogen, CA 19‐9, and CEA assays was evaluated separately using the area under the curve (AUC) value, and the 95% confidence interval (CI) was computed using a standard normal distribution. To determine the ability of GASTROClear to discriminate between various types of GCs, we used the DeLong test to compare the AUC of GASTROClear in different situations [14]. For the cutoff analysis, a cutoff value of 40 was predefined in the IFU of GASTROClear. The adjusted cutoff value (37) for this cohort was defined based on the optimal Youden index. A logistic regression model with fivefold cross‐validation was used to assess the diagnostic performance of GASTROClear in combination with other serum markers for GC detection. The mean AUC values of the fivefold cross‐validated AUC estimates served as performance indicators, and the 95% CI was computed based on the influence curve. All statistical analyses were performed using the R software (version 4.2.2) [15].

## 3 Results

### 3.1 Study Participant Characteristics

**FIGURE 1:**
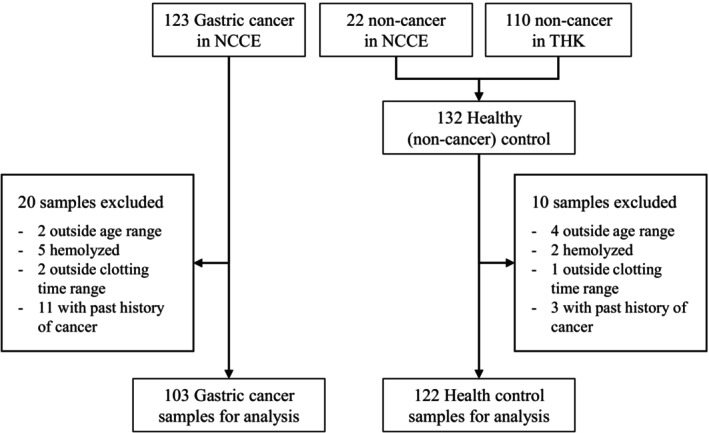
Flowchart displaying the methods used to select participants for analysis. Blood samples from patients with gastric cancer were collected at the National Cancer Center Hospital East (NCCE), and samples from healthy controls were collected at Tsujinaka Hospital Kashiwanoha (THK) and NCCE.

The final sample size of 103 patients with GC and 122 HC was subjected to analysis using GASTROClear and the other indicated assays (Figure 1). The clinicopathological characteristics of the analyzed samples are shown in Table 1. Patients with GC were 70.2 years old on average, and 64% were positive for Hp‐Ab, whereas HC participants were 52.7 years old on average, and 20% were positive for Hp‐Ab. The difference in age distribution between patients with GC and HC in our sample set was consistent with the characteristic age difference observed for GC [16] and the population undergoing mass screening for GC [17]. Similarly, the difference in the Hp‐Ab positivity rate reflected a positive correlation between GC and Hp infection [18], although the positivity rate for both groups was lower than that previously reported [19]. This was presumably because a substantial number of GC (33%) and HC (10%) participants had a history of Hp eradication therapy (Table 1). The groups were well balanced regarding sex distribution (69% of the patients with GC and 66% of the HC participants were men) and body mass index (an average of 22.5 kg/m^2^ for patients with GC and 22.8 kg/m^2^ for HC participants). Most patients with GC (59%) had no symptoms. Accordingly, 58% of patients with GC were diagnosed at an early stage (Stage I), and 40% underwent endoscopic resection. This is consistent with the previously reported distribution of GC stages diagnosed during mass screening [20]. The median size of the gastric tumors was 30 mm, and the tumors were mainly located in the middle of the stomach (48%). The proportion of histopathological types was similar between differentiated (54%) and undifferentiated (42%) GC.

**TABLE 1:** Clinicopathological characteristics of analyzed participants.

Characteristics	Gastric cancer (*N* = 103)	Healthy control (*N* = 122)
Age (year), average (range)	70.2 (44–87)	52.7 (40–79)
Sex, *n* (%)		
Male	71 (69)	80 (66)
Female	32 (31)	42 (34)
BMI, average (range)	22.5 (13.6–36.5)	22.8 (16.2–30.8)
Symptom, *n* (%)		
Absent	61 (59)	122 (100)
Present	42 (41)	
Atrophic gastritis, *n* (%)		
Absent	4 (4)	81 (66)
Present	99 (96)	41 (34)
Serum anti‐HP IgG antibody, *n* (%)		
Negative	37 (36)	98 (80)
Positive	66 (64)	24 (20)
History of HP eradiation, *n* (%)	34 (33)	12 (10)
Tumor size (mm), median (range)	30 (10–150)	
Location, *n* (%)		
Upper	26 (25)	
Middle	49 (48)	
Lower	28 (27)	
Histopathology type, *n* (%)		
tub1/tub2	56 (54)	
por/sig	43 (42)	
Others	4 (4)	
Stage (TNM), *n* (%)		
Stage I	60 (58)	
Stage II	17 (16)	
Stage III	13 (13)	
Stage IV	13 (13)	
Treatment indication, *n* (%)		
Endoscopic resection	41 (40)	
Surgery	47 (46)	
Chemotherapy	15 (14)	

### 3.2 Diagnostic Performance of GASTROClear for GC

**FIGURE 2:**
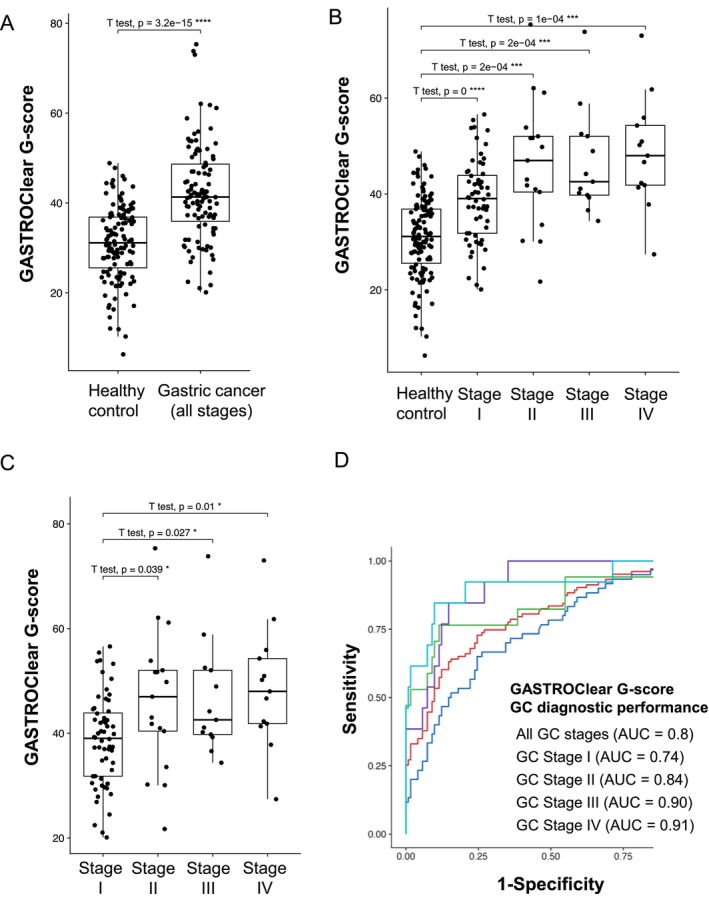
Diagnostic performance of GASTROClear in gastric cancer detection. (A) Box plot analysis of the GASTROClear *G*‐score between healthy control participants and all patients with gastric cancer. (B) Box plot analysis of the GASTROClear *G*‐score for patients with each stage of gastric cancer versus healthy control participants. (C) Box plot analysis of the GASTROClear *G*‐score for patients with GC stage II–IV versus GC stage I. (D) Receiver operating characteristic (ROC) analysis for evaluating the diagnostic value of GASTROClear for gastric cancer detection (including data for all stages and each stage separately).

**FIGURE 3:**
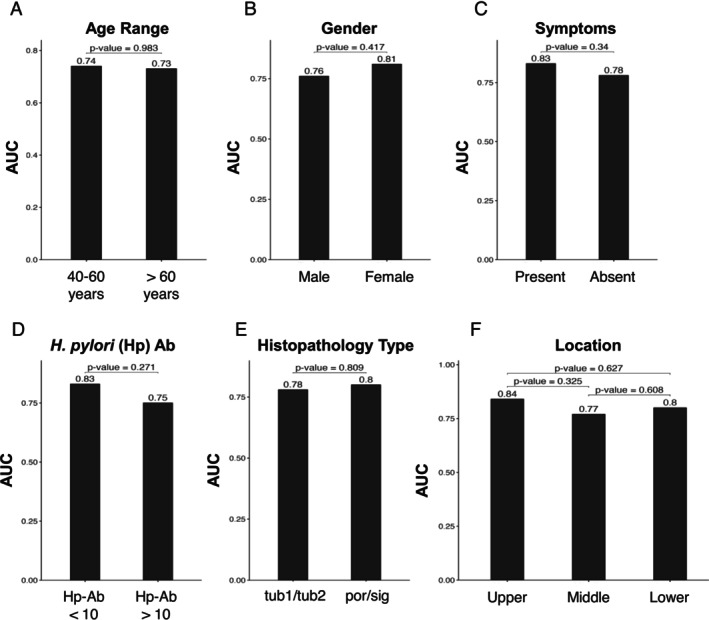
Diagnostic comparison of GASTROClear based on the clinicopathological characteristics of patients with gastric cancer. AUC comparison of the G‐score was analyzed according to (A) age range with 60 years as the cutoff, (B) sex, (C) presence of symptoms, (D) Hp‐Ab value with 10 as the cutoff, (E) histopathology type, and (F) location of gastric cancer.

The diagnostic performance of GASTROClear for detecting GC is shown in Figure 2. The *G*‐scores of all GC samples were significantly higher (median 42.0) than those of the HC samples (median 30.9, *p* = 3.2e^−15^) (Figure 2A). The median G‐scores for each GC stage were 39.0 (Stage I), 47.0 (Stage II), 42.6 (Stage III), and 48.0 (Stage IV). These were all significantly higher than those for HC (Figure 2B). As expected, the median G‐scores for GC stage II‐IV samples were also significantly higher than that for GC stage I samples (Figure 2C). The AUC of the G‐score for all GC samples was 0.80 (CI 0.73–0.85) (Figure 2D). The AUCs for Stages I, II, III, and IV were 0.74, 0.84, 0.90, and 0.91, respectively (Figure 2D). The diagnostic performance of GASTROClear based on clinicopathological characteristics is shown in Figure 3. No significant difference in AUC was observed by age, sex, presence of symptoms, Hp‐Ab value, histological type, and tumor location when using the cutoff values of 60 years (age) and 10 (Hp‐Ab value).

### 3.3 Sensitivity and Specificity of GASTROClear With a Predefined and Adjusted Cutoff Point

With a predefined risk score cutoff value of 40, GASTROClear demonstrated a sensitivity of 56.9% and specificity of 87.6%. When GCs were stratified into early‐stage (Stage I) and advanced‐stage (Stage II–IV), the sensitivity was 43.3% for Stage I and 76.2% for Stages II–IV (Table 2). To further understand the performance range of the predefined cutoff, we re‐evaluated the G‐score in our Japanese cohort and identified an optimal cutoff value of 37. This value accurately predicted GC with a sensitivity of 70.9% and specificity of 75.4% for all stages of GC and a sensitivity of 61.7% for Stage I and 83.7% for Stages II–IV (Table 2).

**TABLE 2:** Sensitivity and specificity of GASTROClear with predefined and adjusted cutoff scores.

GC stage	Sensitivity (%)	Specificity (%)
*Predefined cutoff (G score of 40)*		
All stages	57.3	87.7
Stage I	43.3	
Stages II–IV	76.7	
*Adjusted cutoff (G score of 37)*		
All stages	70.9	75.4
Stage I	61.7	
Stages II–IV	83.7	

### 3.4 Diagnostic Performance of GASTROClear in Combination With Other Serum Markers

**FIGURE 4:**
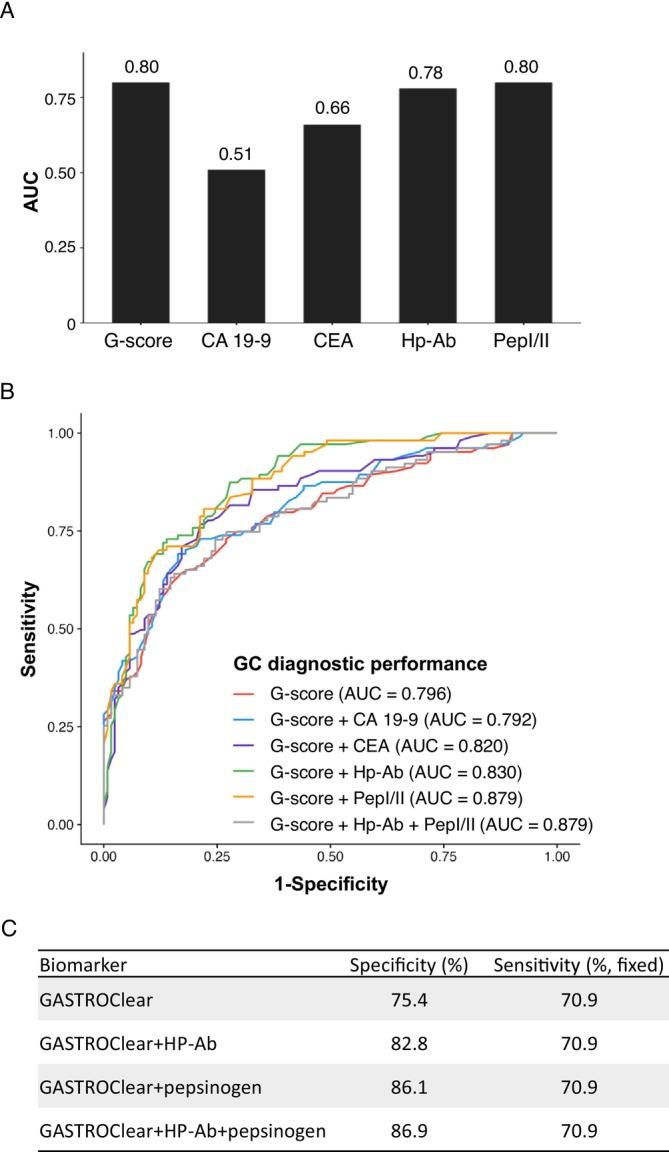
Diagnostic performance of GASTROClear in combination with other serum markers. (A) Comparison of the diagnostic performance of GASTROClear to other serum markers. (B) AUC comparison between GASTROClear alone and GASTROClear in combination with other serum markers. (C) Performance comparison between GASTROClear and alternative models at a fixed sensitivity.

We assessed the diagnostic capability of other serum biomarkers for GC (Figure 4A). GC develops against a background of atrophic gastritis caused by *H. pylori* ; therefore, Hp‐Ab and pepsinogen yielded comparable AUC values of 0.78 and 0.80, respectively, to that of the GASTROClear G‐score. In contrast, the diagnostic performances of CA 19‐9 and CEA were inferior to those of GASTROClear, with AUC values of 0.51 and 0.66, respectively. Patients who have received previous Hp eradication therapy are typically excluded from Hp‐Ab testing as diagnostic performance is poorer in these patients. In our study, there was no significant difference in the diagnostic performance of GASTROClear between participants who had received Hp eradication therapy prior and those who had not (Figure S1).

To augment the diagnostic performance of GASTROClear further, we tested whether it could be combined with known serum markers for GC detection (Figure 4B). These novel biomarker combinations were effective for GC diagnosis, with an AUC of 0.830 (*G*‐score + Hp‐Ab) and 0.879 (*G*‐score + pepsinogen), both of which were significantly higher than that of the GASTROClear *G*‐score alone. The other dual combinations were less additive, with an AUC of 0.820 (*G*‐score + CEA) and 0.792 (*G*‐score + CA 19–9). When GASTROClear was combined with both Hp‐Ab and pepsinogen, the AUC value was the same as that of the *G*‐score of the pepsinogen combination (0.879) (Figure 4B). A direct comparison of cutoff performances relative to GC serum biomarkers was performed using a fixed sensitivity analysis (Figure 4C). We found that the specificity of the GASTROClear *G*‐score alone (75.4%), at a fixed sensitivity of 70.9%, increased to 82.8%, 86.1%, and 86.9% when combined with Hp‐Ab, pepsinogen, and both analytes, respectively.

## 4 Discussion

In this study, we evaluated the diagnostic performance of GASTROClear, a commercialized first‐in‐class serum miRNA biomarker panel for GC detection, in a Japanese cohort and verified the reproducibility of its diagnostic performance. Our data suggest that GASTROClear can effectively diagnose GC with various clinicopathological features and has promising diagnostic performance, even for early‐stage and asymptomatic GC. We also demonstrated that GASTROClear synergizes well with other serum markers, such as Hp‐Ab and pepsinogen, to achieve higher diagnostic efficacy for GC in Japan. Although prior studies, such as those by Abe et al. and Izumi et al. [21, 22], have evaluated the diagnostic utility of serum miRNA biomarkers in GC detection among Japanese populations, our study builds upon these findings by testing a commercially available miRNA panel, GASTROClear.

In countries with a high prevalence of GC, such as Japan and South Korea, public health mass‐screening programs using gastrointestinal endoscopy have been established [3, 4]. However, the effectiveness of population‐wide endoscopic screening may be limited by endoscopic capacity. Screening compliance may also be further limited by the unwillingness of part of the population to undergo an invasive procedure such as endoscopy for screening. Less invasive screening methods such as gastric radiography and serodiagnosis have conventionally been used to identify individuals at high risk of GC so that they can be prioritized for gastric endoscopy [23, 24]. Gastric radiography is routinely performed during medical checkups in Japan. However, its detection rate is only approximately half that of gastric endoscopic screening [7]. Serodiagnosis, or disease stratification by serological tests to detect surrogate GC biomarkers such as Hp‐Ab and pepsinogen, has been deployed with varying degrees of success [25, 26]. However, Hp eradication therapy has been widely implemented and covered by insurance in Japan since 2013. As *H. pylori* infection becomes less prevalent following more widespread Hp eradication therapy, tests like GASTROClear, which can detect GC early regardless of *H. pylori* status, would become more important in the future, since individuals in whom *H. pylori* was eradicated may still have a reasonable risk of GC [27].

In this study, the AUC of GASTROClear was 0.80, which is slightly lower than its previously reported AUC of 0.85 [10]. The discrepancy between the cohort studies in Japan and Singapore may be attributed to (1) the fact that this study enrolled approximately 60% of early‐stage GC cases and (2) the fact that the pathological diagnostic criteria for early‐stage GC differ between the two countries [10]. The TNM staging of GC in this study was based on the UICC classification, whereas the pathological diagnosis in the previous report was based on the American Joint Committee on Cancer (AJCC) classification [28]. Hence, a substantial number of Stage I GC cases in this study would have been diagnosed as high‐grade dysplasia according to the AJCC classification. This assumption is supported by the fact that 40% of the patients with GC in this study were successfully treated with endoscopic treatment (Table 1). In previous reports, the sensitivity to high‐grade dysplasia was 60%, which was lower than that of the other stages but consistent with the sensitivity to Stage I GC by the adjusted cutoff in the current study (Table 2) [10]. Importantly, the AUC value for Stage II–IV GC in this study was reproducible and comparable to those reported in previous studies [10]. For the cutoff analysis, we adjusted the value downward from 40 to 37 despite the reproducibility observed in the AUC values. We speculate that this could be due to differences in the blood collection tubes or the instability of miRNAs in serum samples during the storage and transportation processes. For other blood‐based GC markers, it should be noted that Hp‐Ab and pepsinogen showed significantly lower performances in the Singapore study, whereas their performances greatly increased in this cohort study. This may be because in Japan, owing to the existence of Hp‐Ab and pepsinogen screening programs, patients with positive GC results are more likely to be recruited than in Singapore, where Hp‐Ab and pepsinogen testing are not common or non‐existent.

Based on the findings of this study, we observed the following advantages of GASTROClear: First, GASTROClear is able to detect GC at all stages, including above 60% of stage I GC. Second, *H. pylori* infection status and prior eradication therapy had no significant impact on diagnostic performance. Third, GASTROClear performance, particularly the specificity of the test, is improved when used in combination with other known serum markers. A specificity of 85% or more can be achieved when GASTROClear is combined with Hp‐Ab and pepsinogen. Fourth, when considering the implementation of GASTROClear in clinical practice, it is important to weigh its cost against the benefits it offers compared to traditional gastroscopy. Gastroscopy, while being the gold standard for GC detection with a sensitivity of 95.5% and specificity of 85.1% based on the diagnostic method as reported by Hamajima et al. [29], is an invasive procedure that requires significant resources, including specialized equipment, trained personnel, and patient preparation. In contrast, the GASTROClear panel, being a blood‐based test, is minimally invasive, easier to administer, and potentially more cost‐effective, particularly in settings where gastroscopy resources are limited. The cost per test for GASTROClear is generally lower than that of a gastroscopy, particularly when considering the indirect costs associated with gastroscopy, such as sedation, recovery time, potential complications and human resources. In this sense, GASTROClear may be beneficial as an initial screening tool to identify high‐risk individuals who would benefit most from subsequent gastroscopy. This stratified approach could optimize the use of gastroscopy by targeting it toward those with a higher likelihood of having GC, thereby potentially reducing overall healthcare costs while maintaining high diagnostic accuracy. Finally, GASTROClear may prove particularly useful in specific groups where regular endoscopy is challenging. For instance, in geographically isolated regions such as island areas or for individuals engaged in occupations like pelagic fishing, access to endoscopic facilities may be limited. Similarly, patients who have undergone gastric surgery for obesity may face difficulties in undergoing endoscopic procedures due to anatomical challenges. In these cases, GASTROClear offers a less invasive, more accessible option for GC screening, allowing for earlier detection in populations that might otherwise be underserved.

This study has a few limitations that must be addressed. First, we only collected serum samples from two hospitals. It would be ideal to verify whether data reproducibility can be obtained by including more participants from institutions in other regions of Japan. Secondly, advanced cancers may have been overlooked. Additionally, the sensitivity of GASTROClear to diagnose GC patients with Stage I disease was limited, which may reduce its utility for early‐stage detection. These limitations highlight the potential risk of missing both advanced and early‐stage GC cases, necessitating further refinement of the assay. Although GASTROClear is a feasible screening method, it does not replace existing imaging modalities. Instead, GASTROClear serves as an effective complementary tool. Third, this study did not collect information regarding the presence of simple gastritis among healthy individuals. As a result, we could not evaluate whether the test accurately distinguishes between non‐cancerous benign gastritis and gastric cancer. Future studies should assess the specificity of GASTROClear in differentiating gastric cancer from benign conditions, such as gastritis, to address this limitation. Finally, this is a cross‐sectional study that does not examine whether the risk score in GASTROClear is correlated with current GC prevalence or if it defines future GC risk. Hence, future long‐term observational studies should consider the correlation between the risk score in GASTROClear and the future onset of GC and recurrence after treatment.

In conclusion, this is the first study to evaluate the diagnostic performance of GASTROClear in asymptomatic patients with GC in Japan, where mass screening for GC is common. Our data demonstrate that GASTROClear is a potentially useful serodiagnostic tool that contributes to the risk stratification of GC, particularly when used in combination with existing *H. pylori* infection diagnostics.

## Author Contributions

**Tatsuro Murano:** conceptualization (lead), investigation (lead), writing – original draft (lead). **Ka Yan Chung:** formal analysis (lead), writing – review and editing (equal). **Yew Chung Tang:** conceptualization (equal), writing – review and editing (equal). **Yuki Kano:** investigation (equal), writing – review and editing (equal). **Ken Takeuchi:** investigation (equal), writing – review and editing (equal). **Naoya Sakamoto:** investigation (equal), writing – review and editing (equal). **Takeshi Kuwata:** investigation (equal), writing – review and editing (equal). **Yuan Gao:** formal analysis (equal), writing – review and editing (equal). **Jit Kong Cheong:** supervision (equal), writing – review and editing (equal). **He Cheng:** supervision (equal), writing – review and editing (equal). **Lihan Zhou:** supervision (equal), writing – review and editing (equal). **Tomonori Yano:** conceptualization (equal), supervision (lead), writing – review and editing (equal).

## Ethics Statement

Approval of the research protocol by an Institutional Reviewer Board: The study protocol was approved by the respective institutional review boards of each hospital (2019‐161 at the National Cancer Center Hospital East and 2020‐5 at Tsujinaka Hospital Kashiwanoha).

## Consent

All the participants provided written informed consent.

## Conflicts of Interest

L.Z. is the co‐founder and shareholder of MiRXES. K.Y.C., Y.G., Y.C.T., H.C., and L.Z. are employees of MiRXES. J.K.C. is partially supported by MiRXES through a manpower secondment scheme from the National University of Singapore. Other authors have no competing interests to declare that are relevant to the content of this article.

## Supporting information


Figure S1.

Table S1.

## Data Availability

Data, analytic methods, and study materials will not be made available to other researchers.
