# Supplementary figures and images for: Novel and Effective Blood‐Based miRNA Diagnostic Panel for Gastric Cancer: A Pilot Study in a Japanese Population
Source: Cancer Med. 2025 Apr 18;14(8):e70790. doi: 10.1002/cam4.70790 (PMC12007419; doi:10.1002/cam4.70790)

Supplemental Figure S1

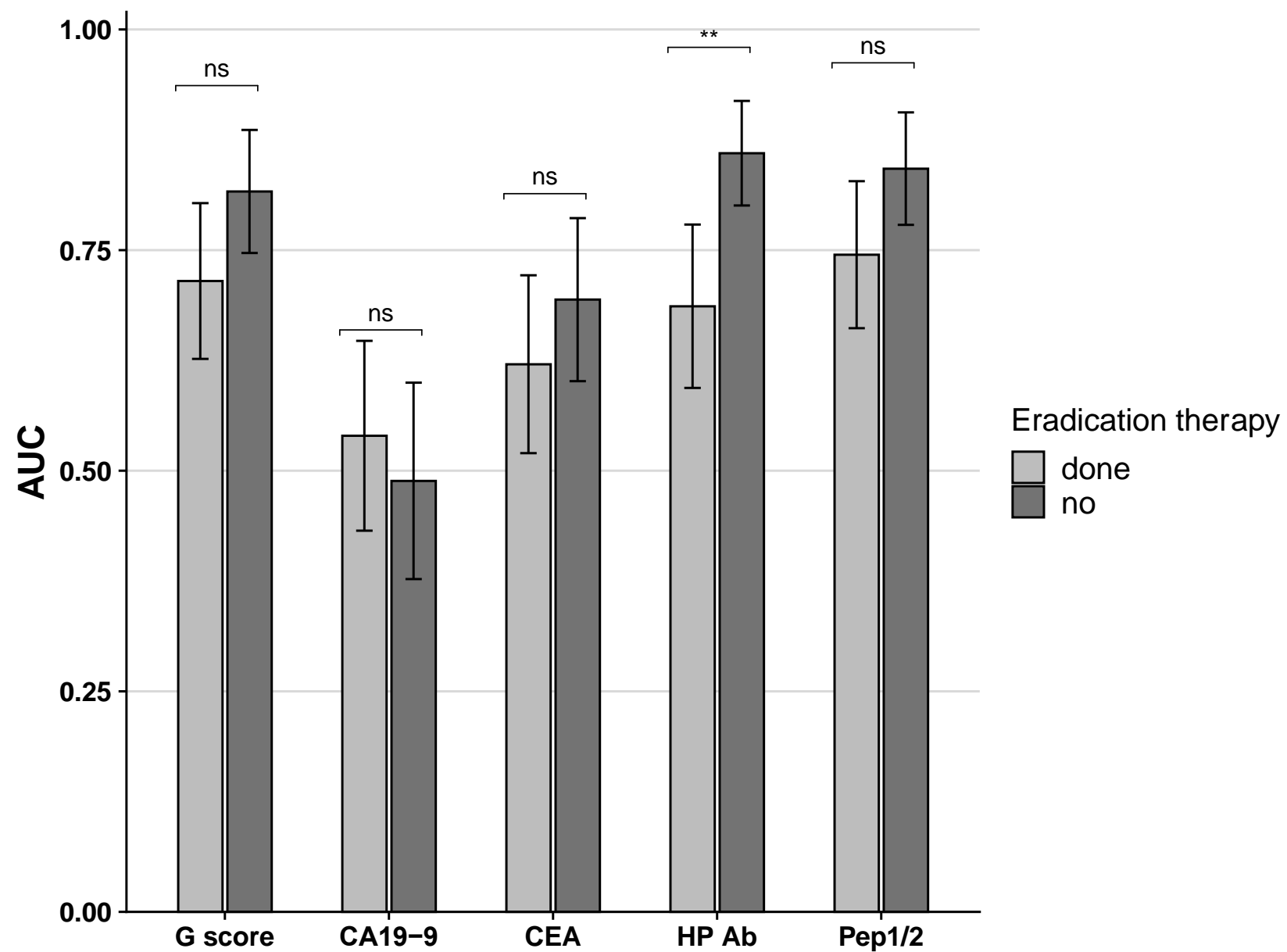

Supplement: Supplementary file 1 — Figure S1. [file CAM4-14-e70790-s001.pdf]
